# EMT is the dominant program in human colon cancer

**DOI:** 10.1186/1755-8794-4-9

**Published:** 2011-01-20

**Authors:** Andre Loboda, Michael V Nebozhyn, James W Watters, Carolyne A Buser, Peter Martin Shaw, Pearl S Huang, Laura Van't Veer, Rob AEM Tollenaar, David B Jackson, Deepak Agrawal, Hongyue Dai, Timothy J Yeatman

**Affiliations:** 1 Merck, Sharp and Dohme, P.O. Box 4, 770 Sumneytown Pike, Building 53, West Point, PA 19486, USA; 2 Merck, Sharp and Dohme, P.O. Box 4, 770 Sumneytown Pike, WP53B-120, West Point, PA 19486, USA; 3 Merck Research Laboratories, UG-4C74, 351 N. Sumneytown Pike, North Wales, PA 19454-2505, USA; 4 Merck, Sharp and Dohme, 33 Avenue Louis Pasteur, Boston, MA 02115, USA; 5 Moffitt Cancer Center, 12902 Magnolia Drive, Tampa, FL 33612, USA; 6 LIFE Biosystems GmbH, Poststrasse 34, D-69115 Heidelberg, Germany; 7 Diagnostic Oncology, The Netherlands Cancer Institute, Plesmanlaan 121, 1066 CX, Amsterdam, The Netherlands; 8 Leiden University Medical Center, PO Box 9600 2300 RC Leiden, The Netherlands

## Abstract

**Background:**

Colon cancer has been classically described by clinicopathologic features that permit the prediction of outcome only after surgical resection and staging.

**Methods:**

We performed an *unsupervised *analysis of microarray data from 326 colon cancers to identify the first principal component (PC1) of the most variable set of genes. PC1 deciphered two primary, *intrinsic *molecular subtypes of colon cancer that predicted disease progression and recurrence.

**Results:**

Here we report that the most dominant pattern of intrinsic gene expression in colon cancer (PC1) was tightly correlated (Pearson R = 0.92, P < 10^-135^) with the EMT signature-- both in gene identity and directionality. In a global micro-RNA screen, we further identified the most anti-correlated microRNA with PC1 as MiR200, known to regulate EMT.

**Conclusions:**

These data demonstrate that the biology underpinning the native, molecular classification of human colon cancer--previously thought to be highly heterogeneous-- was clarified through the lens of comprehensive transcriptome analysis.

## Background

Colon cancer has long been postulated to be a molecularly heterogeneous disease. This heterogeneity has been proposed as the reason why it has been difficult to identify unifying molecular hypotheses explaining the biology and behavior of the disease. Molecular profiling of colon cancer has been a relatively effective approach for identifying prognosis of early and intermediate stage disease. We and others have identified biologically complex signatures that affect multiple programs such as adhesion, invasion, and angiogenesis and correlate well with cancer progression and recurrence. These signatures appear to support Weinberg's hypothesis [1] of multiple programs leading to cancer development and progression. These signatures have generally been developed using *supervised *machine learning techniques that train their models on pre-determined good vs. poor prognosis patient populations [2-6]. Colon cancer, unlike breast cancer where luminal and basal "intrinsic" subtypes have been identified [7-13], or bladder cancer where intrinsic signatures of recurrence have been established [14,15], has yet to be classified by *unsupervised*, molecular profiling approaches. We believed it was important to attempt to uncover unbiased, native biological traits that might underpin colon cancer.

## Methods

### Colon Cancer Samples

326 human colon cancer samples derived from the Moffitt Cancer Center were previously assessed using a single Affymetrix U133Plus2.0 platform and single standard operating procedure. Formalin fixed paraffin blocks (FFPE) were obtained for 69 of these cases and used to extract tumor RNA after macrodissection. Tumor RNA was submitted for global microRNA analysis using an Applied Biosystems platform covering ~700 unique microRNA species. The gene expression data were then compared directly to the microRNA data derived from the same samples. All patient samples and clinical information for the 326 colon samples were obtained through a protocol approved by The University of South Florida Institutional Review Board.

### Identification of the cell line derived EMT signature

The EMT signature was derived from a microarray dataset with 93 lung cancer cell lines by performing a t-test comparing cell lines exhibiting mesenchymal-like gene expression pattern (high levels of VIM and low levels of CDH1) vs. cell lines with epithelial-like gene expression pattern (low levels of VIM and high levels of CDH1). Genes with p-value < 0.01 by a t-test were selected, and were split into those that were up-regulated in mesenchymal-like cell lines and those that were up-regulated in epithelial like, and further restricted to approximately 200 unique gene symbols in each up and down regulated gene sets based on the absolute value of the fold change.

### Identification of PC1

Unsupervised analysis of the most variable genes expressed in the colon cancer data set (n = 326) was undertaken to discover new, "intrinsic" biology of colon cancer. Principal component analysis on the entire gene expression data set of 326 CRC samples, as implemented in the Princomp function in Matlab, (Mathworks Inc.), was computed by selecting the 1st principal component (PC1) corresponding to the highest eigenvalue of the covariance matrix, describing the inherent variability of the data.

### Derivation of colon signatures

We identified a set of gene sets that were associated with different endpoints related to tumor histology. Signatures for each of the following scenarios was created: right/left (RT/LT) colon was computed by comparing 60 samples collected in RT Colon vs. 18 samples collected in LT Colon; Mucinous/Non-Mucinous colon carcinoma was developed by comparing 35 mucinous colon carcinomas vs. 165 non-mucinous; MSI/MSS was created by comparing 6 MSI vs. 73 MSS samples; Carcinoma vs. Adenoma was developed by comparing 22 pure adenocarcinoma samples vs. 5 pure adenomas; Poor/Well differentiation was discovered by comparing 32 poorly differentiated samples vs. 19 well differentiated, Colon/Rectum by comparing 50 samples collected in colon vs. 19 samples collected in rectum; Stage2/Stage1 was identified by comparing 59 stage 2 samples vs. 32 stage 1 samples, Stage3/Stage2 (71 Stage3 samples vs. 59 Stage2 samples) was similarly identified. Each comparison was carried on non-metastatic samples with known stage, histology, and collection site. For each comparison, two gene sets (up and down regulated) were identified by t-test with p-value < 0.01, split by a sign of fold change, selection of unique gene symbols among 100 probes most differentially expressed by an absolute value of fold change. Performance of these gene sets was evaluated by back substitution and the scores for gene sets were computed as the mean of probes mapped by the gene symbol to the up-regulated subset minus the mean of the probes that mapped by the gene symbol to the down-regulated subset. They were found to have ROC AUC>0.7 and 1-way ANOVA p-value < 1e-6 when applied to distinguish the same samples that were used to identify these gene sets.

### Scoring of signatures in the data set

Signature score for a given gene set was obtained by averaging the expression levels of the probes that mapped by the gene symbol to that gene set. MYC and RAS signatures were obtained from Nevins et al [16,17].

### Standard microarray data processing

The microarray data was processed by running RMA normalization method as implemented in Affymetrix Power Tools using default settings, background correction and quantile normalization with subsequent application of log10 to obtained probe intensities.

## Results

**Figure 1:**
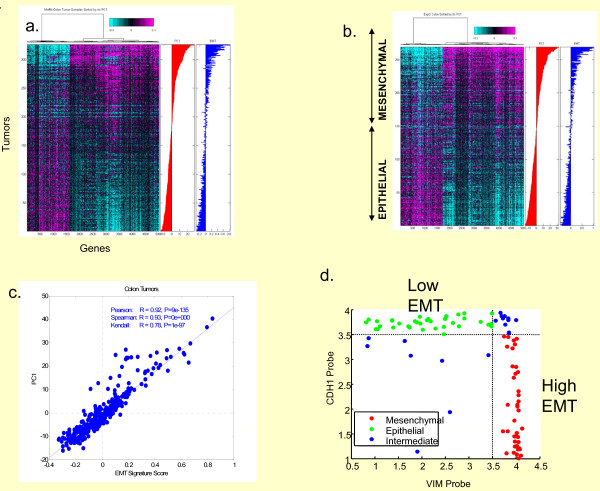
**Intrinsic molecular stratification of human colorectal cancer**. *Unsupervised *analysis and hierarchical clustering of global gene expression data derived from colorectal cancer cases identified 2 major "intrinsic" subclasses (cyan and magenta) distinguished by the first principal component (PC1) of the most variable genes. These two key native subtypes were clearly identified in both the (a) Moffitt Cancer Center (MCC) data set (n = 326) and the (b) EXPO dataset (n = 269). PC1 was later found to be tightly correlated with an EMT signature derived from cell lines, providing an explanation for the biology underpinning these two intrinsic classes in both datasets. PC1 clearly distinguishes two subclasses which were subsequently identified as epithelial vs. mesenchymal. On both panels (a) and (b), mean-centered probe intensities are shown, and probes are clustered using Pearson correlation based distance and Ward linkage. Also, rows represent samples, and columns represent array probes. Panel (c) shows scatter plot of EMT signature score and PC1 (First Principal Component Score) on Moffitt Cancer Center data set. Panel (D) shows the scatter plot between probe intensities for Vimentin (VIM) and E-cadherin probes in a panel of 93 Lung Cancer Cell Lines. Cell Lines exhibiting epithelial-like phenotype are shown in green; those exhibiting mesenchymal-like phenotype are shown in red.

We took a completely *unsupervised *approach to classifying a set of 326 colon and rectal cancers from a spectrum of clinical stages. We set out to identify the most differentially expressed genes, and used the first principal component (PC1) (~5000 differentially-expressed genes) to describe two major subpopulations (Figure 1a, b). The biology of the ~5000 genes underpinning the "intrinsic" PC1 signature was not forthcoming from the standard functional analyses algorithms that often identify multiple pathways linked to complex signatures. In fact, analysis of PC1 by Ingenuity, Kegg, and GeneGo approaches identified multiple potential pathways that might be responsible for the observed molecular subclassification (Additional File 1). This approach did not precisely clarify the biology behind the observed gene expression changes, but suggested that adhesion and extracellular matrix were significantly affected. To better describe the functionality of PC1, we examined numerous (~300) cell line-derived and tumor-derived signatures for their association with PC1. This analysis identified the cell line derived epithelial-mesenchymal transition gene expression signature as the most significantly associated (P < 10^-135^) with PC1 (Figure 1c). The signature was derived from an analysis of 93 lung cancer cell lines that had been previously globally molecularly profiled and sorted by two genes associated with the EMT phenotype, CDH1 and VIM (Figure 1d, Additional File 2). The cell lines were then divided into two groups, one considered to be *epithelial *(high CDH1, low VIM) and one considered *mesenchymal *(low CDH1, high VIM) (Additional File 3). The two groups were then used to identify ~300 genes that would further distinguish epithelial from mesenchymal cell lines. This group of genes then became a cell line derived "EMT signature". More importantly, the up and down arms of the EMT signature were directionally correlated with PC1 (P < 10^-16^, Fisher Exact Test) (Additional File 4). The significant finding was that the *unsupervised *PC1 signature, which represented an "intrinsic" subtype classifier of colon cancer, appeared to be driven by a core EMT program of up-and down-regulated genes (Additional File 4). In fact, 92% of probes mapped to EMT UP gene set (genes that were up-regulated in mesenchymal vs. epithelial lung cell lines) were positively correlated with PC1 and 82% of probes from EMT DOWN gene set (genes that were respectively down-regulated), corresponding to Fisher exact test p-value of 2 × 10^-16 ^[18].

**Figure 2:**
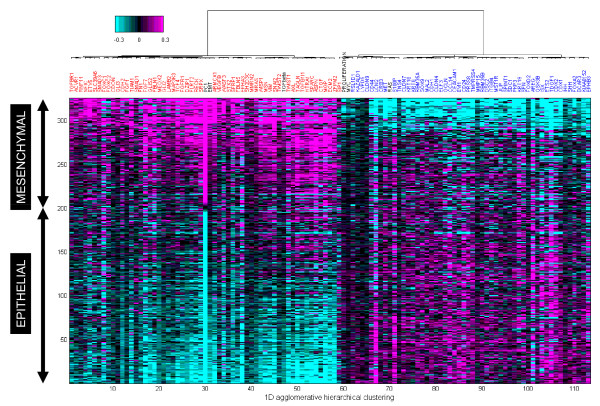
**Hierarchical cluster analysis of the top 100 genes assessed from a text mining approach were strongly associated with the EMT program as shown on 326 MCC colon tumors sorted by PC1**. The 100 gene set contains individual genes (CDH1, CLDN9, FGFR1, FN1, TWIST 1 & 2, AXL, VIM) as well as signatures of genes (PC1, EMT, TGFbeta, Proliferation, MYC, and RAS) that are up-regulated in mesenchymal tumors (shown in magenta), and that are up-regulated in epithelial tumors (shown in cyan). Names for the relevant gene signatures are shown in black. Samples (rows) are sorted by PC1. Genes (columns) are clustered using Pearson correlation and Ward linkage. Heatmap shows mean-centered probe intensities.

We further confirmed the expression of this same embedded pattern of gene expression (PC1) in the independent ExPO data set (n = 269) (Figure 1b), suggesting that EMT is a pervasive program underpinning colon cancer biology. To further clarify the EMT association with the PC1 signature, genes previously linked to the EMT program such as VIM, FGFR, FLT1, FN1, TWIST, AXL, and TCF, were individually assessed and found to be positively correlated with PC1/EMT (Figure 2). Similarly, genes such as CDH1, CLDN9, EGFR, and MET were negatively correlated with PC1/EMT. Also shown are multi-gene *signatures *(black labels) such as EMT, TGF-beta, RAS, proliferation, and MYC; TGF-beta is a known driver of EMT and thus correlates with both PC1 and EMT. Alternatively, RAS activation/dependency/addiction has been shown to anti-correlate with EMT [19]. K-RAS dependent cells exhibit an epithelial morphology, expressing significant cortical CDH1 but little VIM. Conversely, RAS-independent cells express little CDH1 but significant VIM. Our results are consistent with these findings. Of interest, proliferation, and an effecter of such (MYC), both anti-correlate with EMT.

**Figure 3:**
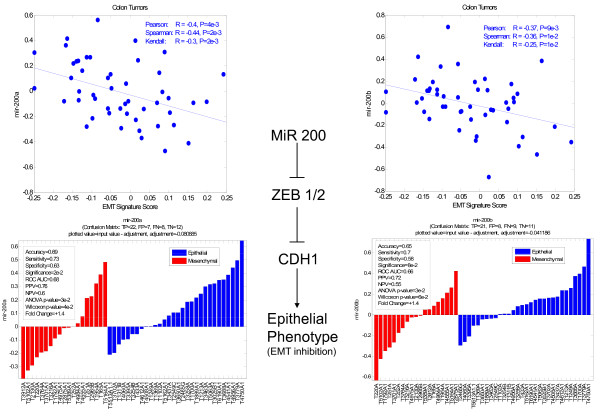
**Correlation of microRNA analysis (~700) with PC1/EMT across 49 colorectal cancers identified the MiR200 family as strongly, negatively correlated with PC1/EMT (upper plots)**. The Mir 200 family has been linked to *inhibition *of EMT (promotion of the epithelial phenotype) through *inhibition *of Zeb 1 & 2, known transcriptional repressors of CDH1. Waterfall plots show MiR 200 over-expression is correlated with more tumors classified as epithelial than mesenchymal and Mir 200 under-expression is correlated with fewer epithelial than mesenchymal tumors (lower plots).

To determine if the EMT signature might be regulated by specific microRNAs [20], we re-profiled ~70 Stage I-IV colon cancers with a ~415 global MiR platform that had been previously assessed by microarray analysis. Out of these ~70 samples, 49 were subsequently used for the analysis after data processing and QC. Of all the MiRs tested, the MiR 200 family was the most highly anti-correlated with PC1/EMT signatures (Figure 3, Additional File 5, Additional File 6). Whereas the gene expression analysis to derive PC1 was performed from frozen tissues, the MiR analyses were performed using matched formalin-fixed, paraffin-embedded tissues (FFPE), strengthening the observed finding *across *platforms.

**Figure 4:**
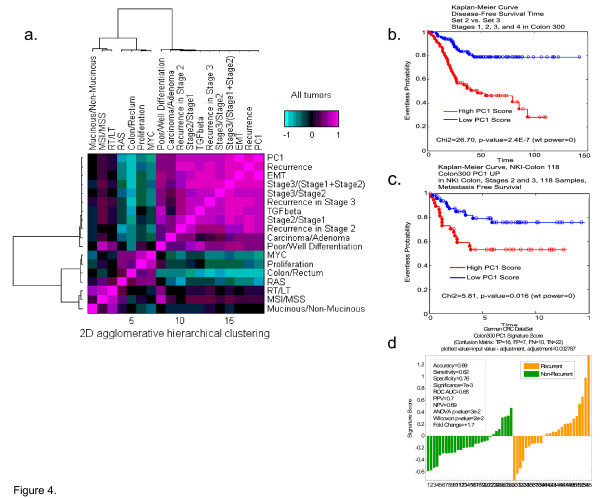
**Covariance matrix showing correlation of PC1 with disease recurrence**. (a) PC1, despite being developed with unsupervised approaches, appeared to correlate well with EMT, disease recurrence, disease progression, and differentiation status, but not with gene signatures linked to adenoma vs. carcinoma, MSI status, or mucinous vs. nonmucinous cancers. Moreover, PC1 appeared to be anti-correlated with RAS, MYC, Proliferation, and colon laterality. PC1 distinguishes good and poor prognosis patients in the MCC data set (b) as well as in two independent test sets: (c) Netherlands Cancer Institute (NKI, #13) (L.V.V.) and (d) Lin et al data sets [21].

Having identified PC1 as an intrinsic gene expression signature closely linked to the EMT program, we wanted to determine if the *mesenchymal *phenotype (high PC1/EMT score) would predict recurrence of disease. To our surprise, PC1, despite being developed with an unsupervised approach, was capable of differentiating good from poor prognosis, and was well-correlated with recurrence and progression of disease, even for intermediate stages II and Stage III (Figure 4a, Additional File 7, 8, 9, 10). It was also linked to cancer progression and to poor differentiation status. We further identified these relationships in the Lin et al [21], NKI, and EXPO data sets (Additional File 11). Moreover, PC1 was also predictive of disease-free survival in the Moffitt Cancer Center colon cancer data set (Figure 4b). More importantly, however, PC1 was also predictive of poor outcome in two additional completely *independent *data sets. In a data set from the NKI, PC1 predicted metastasis free survival (Figure 4c) and in a Lin et al data set [21], PC1 predicted recurrence (Figure 4d). When the PC1 signature is applied to cancers with different recurrence rates, there was also a clear difference between colon, lung, and pancreas cancers (colon < lung < pancreas) (Additional Files 12, 13, 14, 15**and **16).

We also tested known signatures, or developed a number of other signatures within the colon cancer dataset, that did not correlate well with PC1. These included a signature predicting MSI status [22], a signature separating right from left colon tumors, a signature separating mucinous from non-mucinous tumors, and a proliferation signature [23] (Figure 4a). The PC1 signature, while discriminating for epithelial vs mesenchymal tumors, may also be used to classify cell lines. This classification undoubtedly will be useful in further analysis of EMT in cancer using these cell lines as models. Understanding which cell lines best represent the epithelial vs the mesenchymal phenotype will help determine the best models for subsequent drug intervention and target validation studies and will allow cell lines to be mapped to human tumors. For this purpose, we have classified numerous colon cell lines using the tumor-derived PC1 signature (Additional File 16).

## Discussion

Colon cancer has heretofore been considered a very heterogeneous disease [24,25]. It has been difficult to identify a unifying biological theme that could be leveraged for therapeutic intervention. Previously identified prognostic signatures have required supervised learning to elucidate predictive gene sets. For the first time, our data suggest that the PC1 signature, discovered through unsupervised approaches, appears to be a native, intrinsic subtype classifier of colon cancer that predicts recurrence, advancing stage, and poor prognosis based on the biology of EMT. Finding the PC1 score was predictive of recurrence for both stages II and III of colon cancer, and its strong relationship to EMT biology, leads to the possibility that this signature might be useful in these stages for discerning responsiveness to adjuvant chemotherapy. Our data suggest the otherwise molecularly and pathologically heterogeneous disease may be resolved into two principal molecular subtypes of colon cancer: *epithelial *or *mesenchymal*.

The identification of the intrinsic EMT program was further supported by additional molecular studies relating global microRNA profiling data to global gene expression datasets. From this analysis, the MiR 200 family and related MiRs were identified as highly negatively correlated with PC1. This finding was significant because the MiR 200 family has been closely linked to the EMT program. It has been previously demonstrated that MiR 200 over-expression may result in inhibition of ZEB1/2, which in turn leads to inhibition of transcriptional repressors of CDH1, thereby permitting the expression of CDH1 and expression of the epithelial phenotype [26,27]. Thus, a negative correlation of MiR200 and the EMT signature promoting a mesenchymal phenotype is consistent. The relationship between MiR 200 and PC1 was strong enough to be detected on a relatively small number of tumors, even when non-mirror image FFPE tissues were used instead of the original frozen specimen, suggesting the EMT program is pervasive throughout the primary tumor. In addition, MiR 141, a MiR 200 family member, was also identified as negatively correlated with EMT, confirming previous observations. Finally, numerous additional MiRs have been identified that have not yet been previously reported to be linked to EMT.

Analysis of PC1 relative to biological programs beyond EMT was also informative. Of interest was that MSI tumors which are generally prone to better prognosis and right sided predisposition, were shown to have relatively low PC1 scores. Consistent with these data, recent studies have supported the hypothesis that MSI tumors are anti-correlated with EMT [28]. Similarly, mucinous tumors have been linked to local more than distant recurrence, thus the finding that these tumors were more epithelial than mesenchymal is biologically consistent. Most interesting was that the proliferation signature, which has been previously used to identify poor prognosis breast and lung cancers, was linked to good prognosis colon tumors, suggesting proliferation may *not *play a critical role in colon cancer progression, yet we know it is important for colon epithelial biology in crypt bases. Consistent with this is the recent observation that colon metastases have a lower proliferative index than primary non-metastatic tumors [29]. One explanation for this observation may be that metastatic lesions may have undergone a mesenchymal to epithelial transition (MET) [30]. Thus, PC1, a harbinger of the transition to the mesenchymal state, is predictive of poor vs good outcome in a number of observed clinical scenarios. These PC1 anti-correlated signatures shed further light on the biology of colon cancer.

Our data also support the concept that the mesenchymal subtype, linked to TGF-B activation, is likely responsible for advancing stage, poor differentiation status, and distant recurrence of disease, but not to RAS, MYC, MSI, or proliferation. RAS activation, linked to the epithelial phenotype driven by genes such as EGFR, appears to be anti-correlated with the PC1/EMT or mesenchymal signature.

Analysis of other cancers such as pancreas and lung demonstrate a spectrum of EMT across diseases (colon < lung < pancreas) that may explain the differential survivability of these cancers (colon > lung > pancreas), variable recurrence rates, and responses to therapy [31]. And, as we have shown, these observations can now be leveraged to classify cell lines for therapeutic intervention modeling. We anticipate the PC1 score may be useful in classifying tumors with a mesenchymal vs. epithelial phenotype that might be sensitive to new classes of drugs such as Src, Notch, and FGFR inhibitors vs EGFR inhibitors, respectively.

## Conclusions

Collectively, our data suggest that the "intrinsic" PC1 signature, underpinned by robust EMT biology, is highly prognostic for colon cancer recurrence, may be useful to subclassify more than one type of cancer, and may provide a means of identifying sensitive subpopulations for the next generation of novel therapeutics.

## Abbreviations

EMT: Epithelial Mesenchymal Transition; PC1: First Principal Component; MET: Mesenchymal Epithelial Transition

## Competing interests

The authors declare that they have no competing interests.

## Authors' contributions

AL, MN, JW, CB, DH performed data analysis, interpreted the results, and created figures for the manuscript. PS and PH provided scientific direction, critical review and data interpretation. LVV and RT provided clinical samples for the test set. DA provided scientific input into the design and analysis of the experiments. AL and TY were the lead authors of the manuscript.

## Pre-publication history

The pre-publication history for this paper can be accessed here:

http://www.biomedcentral.com/1755-8794/4/9/prepub

## Supplementary Material

Additional file 1 **Ingenuity/GO Analysis produced multiple functional categories for PC1 without bringing clarity to the underlying biology**. The table lists top functional gene groups in terms of significance for enrichment of genes from PC1 signature. Both, significance of enrichment p-value,(based on hyper geometric distribution) and Bonferroni-type correction e-value (to account for multiple testing). Gene sets from Ingenuity, KEGG, and GeneGO were included in the analysis. Click here for file

Additional file 2 **EMT signature was derived by comparing gene expression of cell lines sorted into epithelial or mesenchymal like groups based on CDH1 and VIM expression (see Additional Figure **1). The top 200 up and down probes found most significant by ANOVA (P < 0.001) were selected to represent the EMT signature. The EMT signature contains known EMT drivers such as ZEB1 and ZEB2, TCF4, AXL. It also contains markers such as CDH1, CDH3 for epithelial phenotype and VIM, CDH2 and CDH4 for the mesenchymal phenotype. Click here for file

Additional file 3 **miRNA correlation to EMT and RAS signature scores on mean-centered data**. Pearson correlation coefficient and the associated p-value are provided. Click here for file

Additional file 4 **Centered abundances for 49 tumors × 416 MiR detectors**. Click here for file

Additional file 5 **Out of ~300 signatures tested, EMT was the most significantly associated with PC1 in colon (P < 10^-135^)**. More importantly, the up and down arms of the EMT signature were directionally correlated with PC1 (P < 10^-16^, Fisher Exact Test). See Additional File 2 for list of genes. Click here for file

Additional file 6 **Derivation of the EMT signature used to clarify the biology characterizing PC1**. The EMT signature was derived from a global gene expression analysis of 93 lung cancer cell lines first segregated by differential CDH1 and VIM expression. Right panel shows the relationship between EMT signature score and CDH1 probe intensities, the left panel shows the EMT signature score vs. VIM probe intensity. EMT signature is observed to be positively correlated to VIM and anticorrelated to CDH1. Click here for file

Additional file 7 **Waterfall plot of recurrence prediction of PC1 for the MCC colon dataset shows more recurrences with high signature scores than with low signature scores; similarly there fewer recurrences with low signature scores than with high signature scores**. Click here for file

Additional file 8 **Hierarchical cluster analysis showing expression of key genes (red and blue) and gene signatures (black) in the EMT signature for colorectal tumors**. Genes positively correlated with the EMT signature are shown in red and genes negatively correlated with the EMT signature are shown in blue. Click here for file

Additional file 9 **Hierarchical cluster analysis showing expression of key genes (red and blue) and gene signatures (black) in the EMT signature for lung tumors**. Genes positively correlated with the EMT signature are shown in red and genes negatively correlated with the EMT signature are shown in blue. Click here for file

Additional file 10 **Hierarchical cluster analysis showing expression of key genes (red and blue) and gene signatures (black) in the EMT signature for pancreatic tumors**. Genes positively correlated with the EMT signature are shown in red and genes negatively correlated with the EMT signature are shown in blue. Click here for file

Additional file 11 **Waterfall and boxplot analysis's shows a differential EMT score for colon < lung < pancreas following normalization across all samples**. Click here for file

Additional file 12 **Top 5000 most variable genes (columns) on Colon cell lines (rows) sorted by PC1**. PC1 is observed to be positively correlated to EMT signature score and anti-correlated to RAS signature score. Genes are clustered using Pearson correlation distance metric and Ward linkage. Heatmap shows mean-centered probe intensities. Click here for file

Additional file 13 **PC1 predicts recurrence in stages 2 and 3 of colon cancer**. Data is shown for MCC dataset. Click here for file

Additional file 14 **Covariance matrices showing the relationship of PC1 to the same endpoints as shown in Figure **4a**using (a) independent colon dataset **[21]**(b) EXPO dataset, (c) NKI dataset**. Click here for file

Additional file 15 **EMT signature proposed in this paper is predictive of recurrence in stage 2 and stage 3 MCC tumors**. Click here for file

Additional file 16 **EMT signature proposed in this paper is predictive of recurrence when applied to all tumor samples in MCC data set**. Click here for file
