# Supplementary material for: EMT is the dominant program in human colon cancer
Source: BMC Med Genomics. 2011 Jan 20;4:9. doi: 10.1186/1755-8794-4-9 (PMC3032646; doi:10.1186/1755-8794-4-9)
Supplement: Additional file 1 — Ingenuity/GO Analysis produced multiple functional categories for PC1 without bringing clarity to the underlying biology. The table lists top functional gene groups in terms of significance for enrichment of genes from PC1 signature. Both, significance of enrichment p-value,(based on hyper geometric distribution) and Bonferroni-type correction e-value (to account for multiple testing). Gene sets from Ingenuity, KEGG, and GeneGO were included in the analysis. [file 1755-8794-4-9-S1.PDF]

| Keyword | Source | Pvalue | Evalue |
|---|---|---|---|
| extracellular matrix organization and biogenesis | GO_process | 4.E-15 | 3.E-11 |
| cell adhesion | GO_process | 1.E-14 | 9.E-11 |
| biological adhesion | GO_process | 1.E-14 | 9.E-11 |
| system development | GO_process | 2.E-14 | 1.E-10 |
| anatomical structure development | GO_process | 6.E-14 | 4.E-10 |
| organ development | GO_process | 6.E-14 | 4.E-10 |
| multicellular organismal process | GO_process | 2.E-13 | 1.E-09 |
| multicellular organismal development | GO_process | 3.E-13 | 2.E-09 |
| developmental process | GO_process | 3.E-13 | 2.E-09 |
| collagen fibril organization | GO_process | 6.E-13 | 4.E-09 |
| regulation of angiogenesis | GO_process | 7.E-13 | 5.E-09 |
| extracellular structure organization and biogenesis | GO_process | 2.E-12 | 2.E-08 |
| blood vessel morphogenesis | GO_process | 5.E-11 | 3.E-07 |
| blood vessel development | GO_process | 8.E-11 | 5.E-07 |
| vasculature development | GO_process | 8.E-11 | 6.E-07 |
| angiogenesis | GO_process | 1.E-10 | 7.E-07 |
| anatomical structure morphogenesis | GO_process | 1.E-10 | 8.E-07 |
| Cell adhesion_ECM remodeling | GeneGo | 3.E-10 | 2.E-06 |
| anatomical structure formation | GO_process | 3.E-10 | 2.E-06 |
| organ morphogenesis | GO_process | 7.E-10 | 5.E-06 |
| phosphate transport | GO_process | 1.E-09 | 8.E-06 |
| cell migration | GO_process | 3.E-09 | 2.E-05 |
| regulation of cell migration | GO_process | 1.E-08 | 7.E-05 |
| negative regulation of angiogenesis | GO_process | 3.E-08 | 2.E-04 |
| regulation of cell motility | GO_process | 4.E-08 | 3.E-04 |
| regulation of locomotion | GO_process | 5.E-08 | 3.E-04 |
| cell proliferation | GO_process | 5.E-08 | 3.E-04 |
| locomotion | GO_process | 5.E-08 | 4.E-04 |
| ECM-receptor interaction | kegg | 9.E-08 | 6.E-04 |
| cell motility | GO_process | 9.E-08 | 6.E-04 |
| localization of cell | GO_process | 9.E-08 | 7.E-04 |
| regulation of cell proliferation | GO_process | 1.E-07 | 9.E-04 |
| response to wounding | GO_process | 1.E-07 | 1.E-03 |
| regulation of developmental process | GO_process | 2.E-07 | 1.E-03 |
| skeletal development | GO_process | 3.E-07 | 2.E-03 |
| positive regulation of cell migration | GO_process | 3.E-07 | 2.E-03 |
| negative regulation of cell proliferation | GO_process | 1.E-06 | 7.E-03 |
| inorganic anion transport | GO_process | 1.E-06 | 7.E-03 |
| positive regulation of locomotion | GO_process | 1.E-06 | 8.E-03 |
| positive regulation of cell motility | GO_process | 1.E-06 | 8.E-03 |
| localization | GO_process | 1.E-06 | 9.E-03 |
| Focal adhesion | kegg | 2.E-06 | 1.E-02 |
| positive regulation of biological process | GO_process | 3.E-06 | 2.E-02 |
| cell-substrate adhesion | GO_process | 4.E-06 | 3.E-02 |
| response to external stimulus | GO_process | 5.E-06 | 4.E-02 |
| tissue development | GO_process | 5.E-06 | 4.E-02 |
| anion transport | GO_process | 5.E-06 | 4.E-02 |
| regulation of biological process | GO_process | 9.E-06 | 6.E-02 |
| cell-matrix adhesion | GO_process | 1.E-05 | 8.E-02 |

# Additional File 1.
