# Supplementary figures and images for: EMT is the dominant program in human colon cancer
Source: BMC Med Genomics. 2011 Jan 20;4:9. doi: 10.1186/1755-8794-4-9 (PMC3032646; doi:10.1186/1755-8794-4-9)

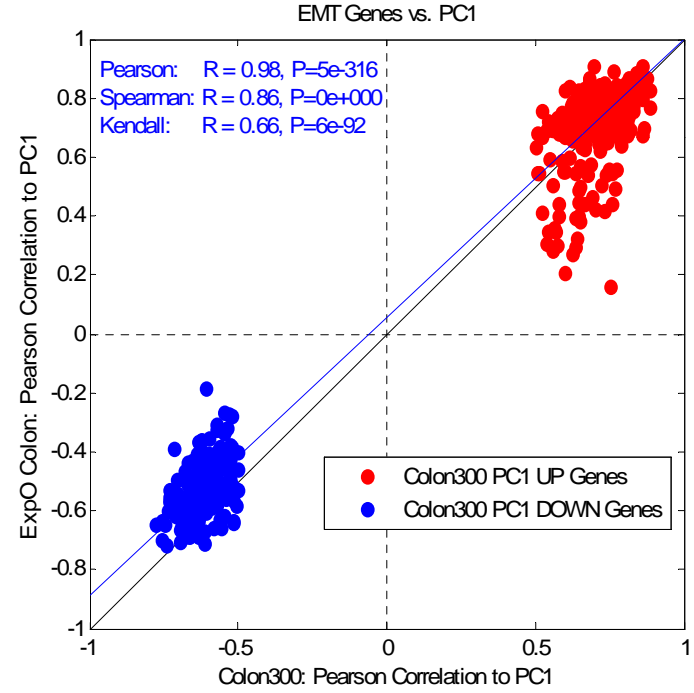

Supplement: Additional file 2 — EMT signature was derived by comparing gene expression of cell lines sorted into epithelial or mesenchymal like groups based on CDH1 and VIM expression (see Additional Figure 1). The top 200 up and down probes found most significant by ANOVA (P < 0.001) were selected to represent the EMT signature. The EMT signature contains known EMT drivers such as ZEB1 and ZEB2, TCF4, AXL. It also contains markers such as CDH1, CDH3 for epithelial phenotype and VIM, CDH2 and CDH4 for the mesenchymal phenotype. [file 1755-8794-4-9-S2.PDF]

Additional **File 3.**

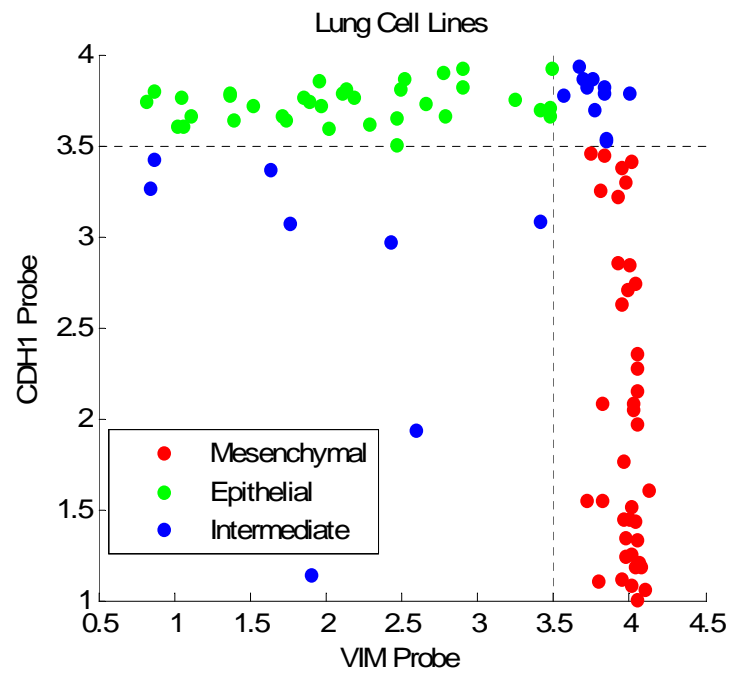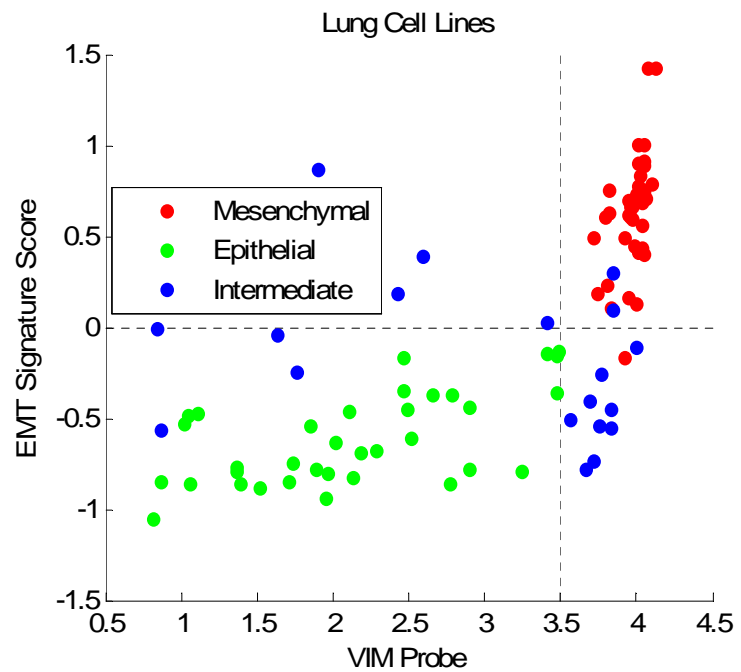

Supplement: Additional file 3 — miRNA correlation to EMT and RAS signature scores on mean-centered data. Pearson correlation coefficient and the associated p-value are provided. [file 1755-8794-4-9-S3.PDF]

A

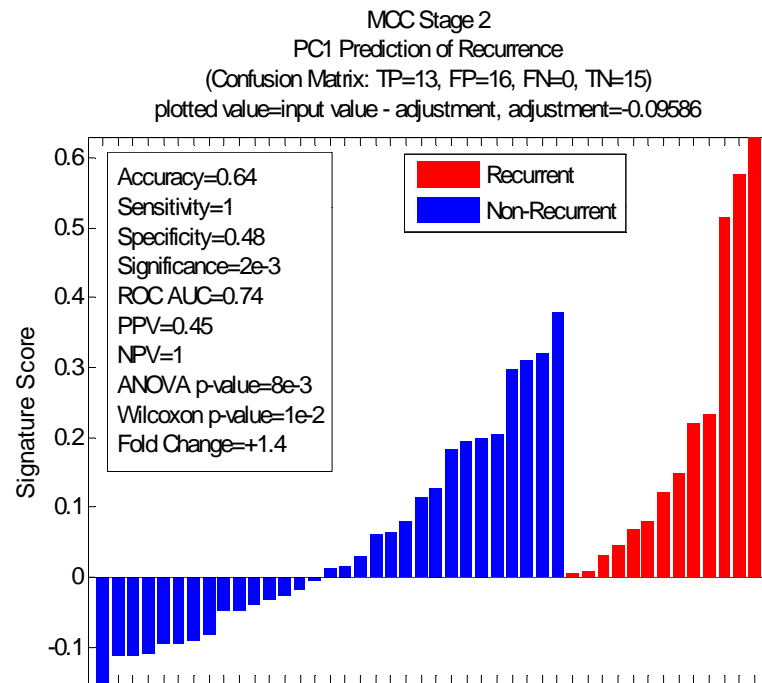

B

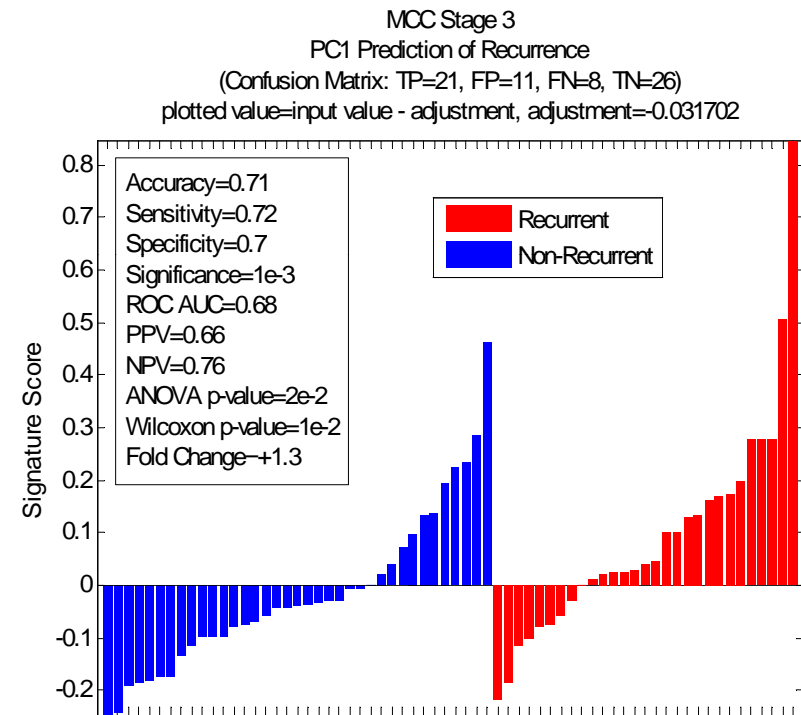

Supplement: Additional file 8 — Hierarchical cluster analysis showing expression of key genes (red and blue) and gene signatures (black) in the EMT signature for colorectal tumors. Genes positively correlated with the EMT signature are shown in red and genes negatively correlated with the EMT signature are shown in blue. [file 1755-8794-4-9-S8.PDF]

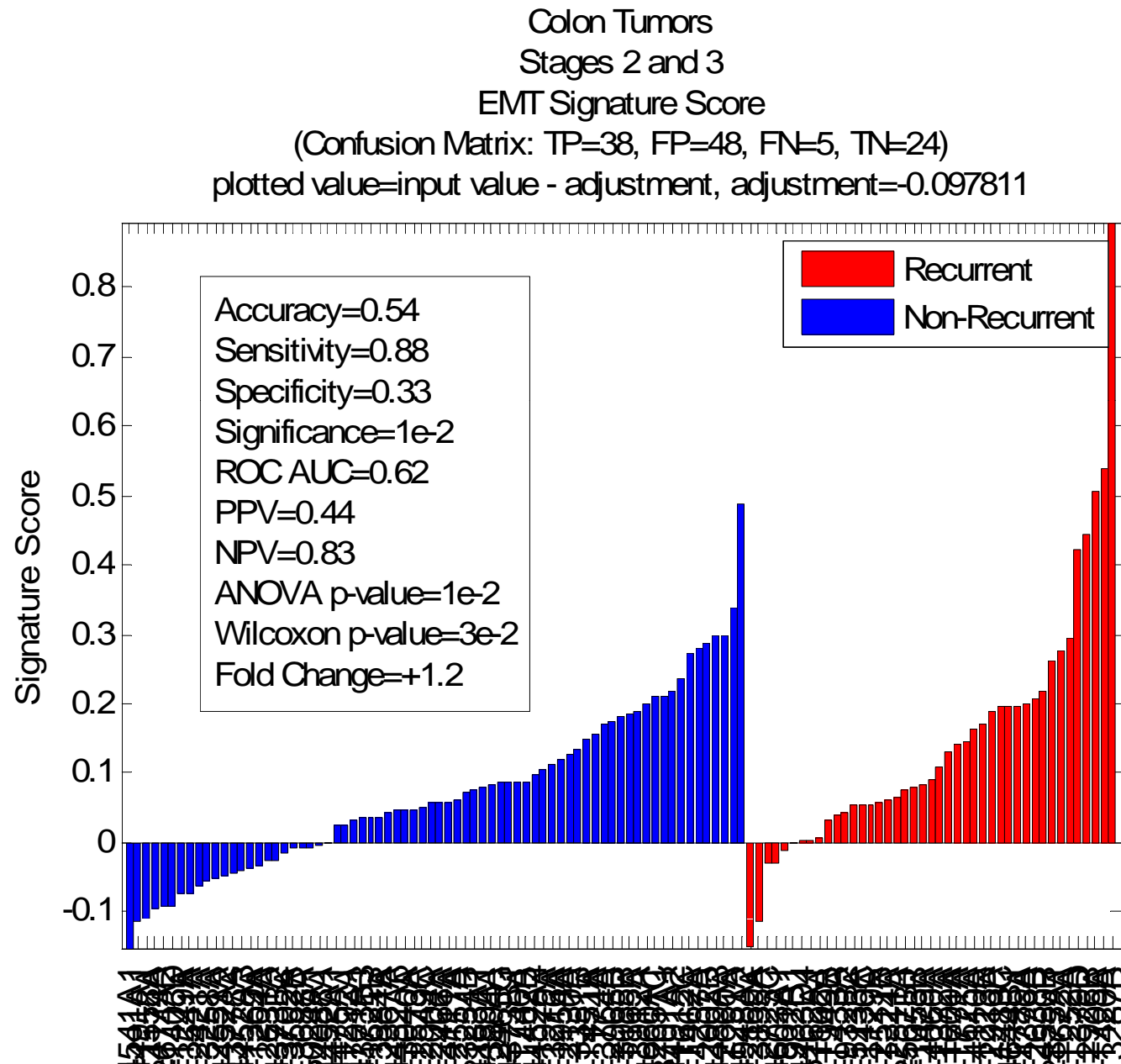

Supplement: Additional file 9 — Hierarchical cluster analysis showing expression of key genes (red and blue) and gene signatures (black) in the EMT signature for lung tumors. Genes positively correlated with the EMT signature are shown in red and genes negatively correlated with the EMT signature are shown in blue. [file 1755-8794-4-9-S9.PDF]

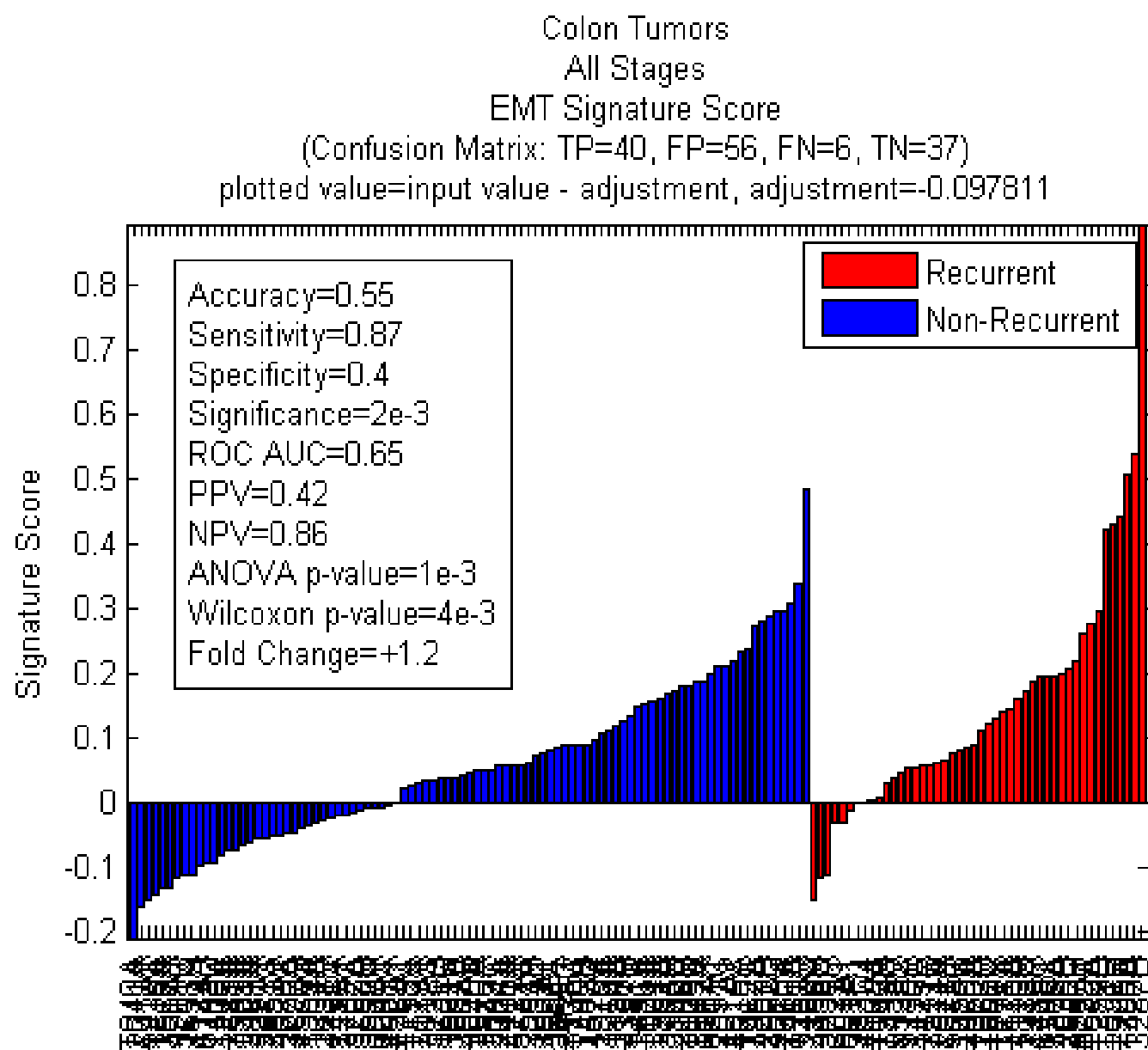

Supplement: Additional file 10 — Hierarchical cluster analysis showing expression of key genes (red and blue) and gene signatures (black) in the EMT signature for pancreatic tumors. Genes positively correlated with the EMT signature are shown in red and genes negatively correlated with the EMT signature are shown in blue. [file 1755-8794-4-9-S10.PDF]

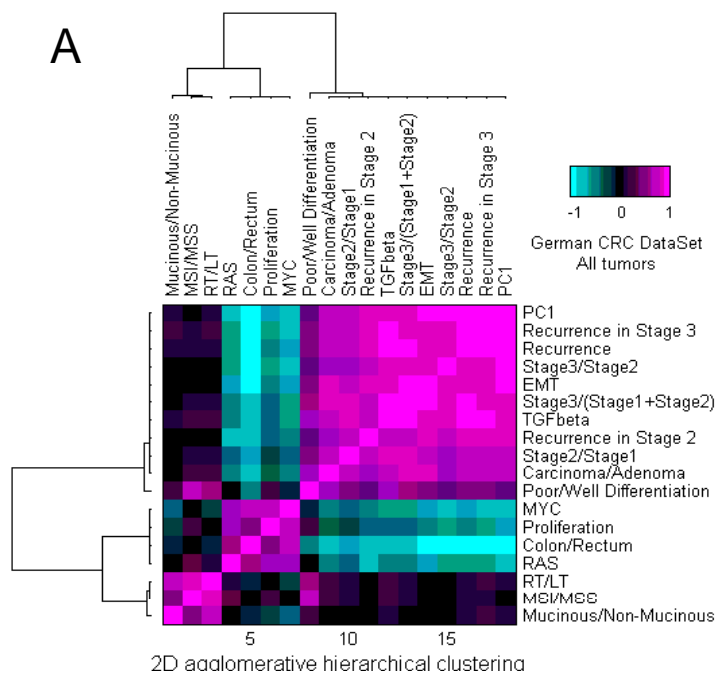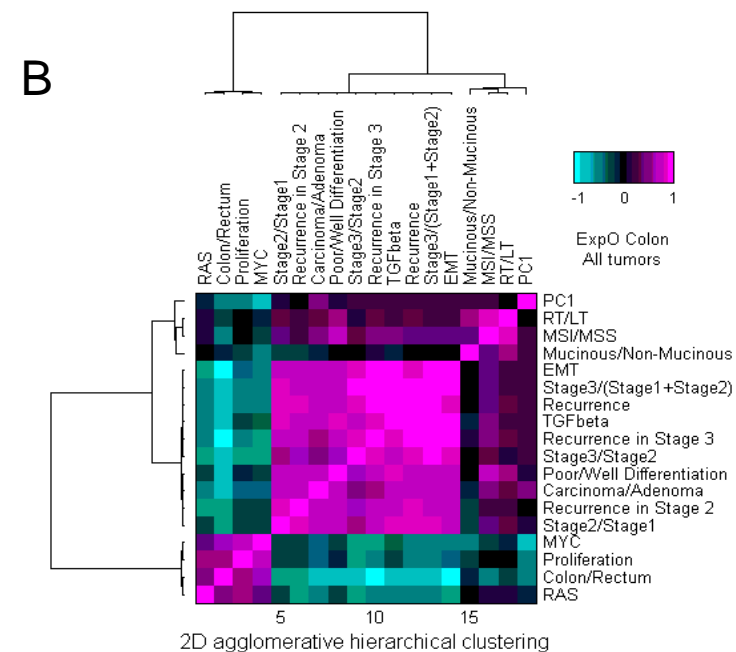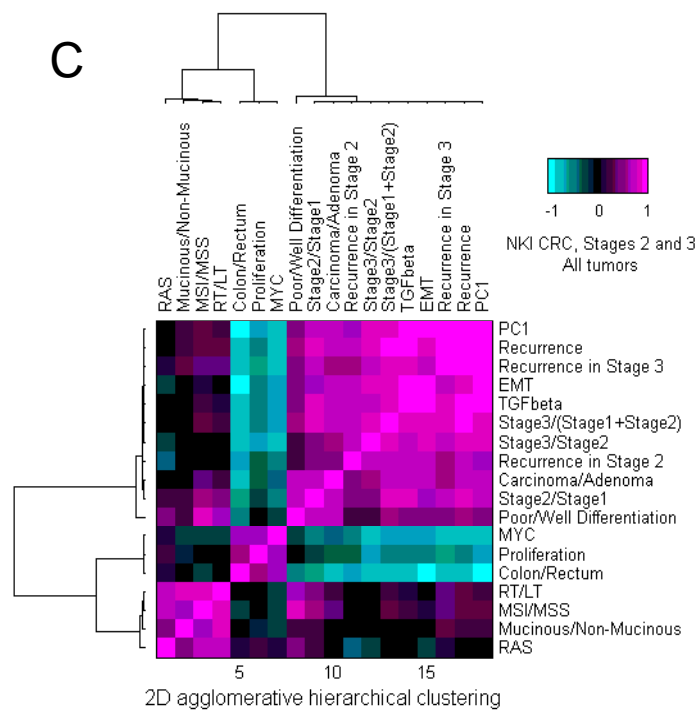

Additional File 11:

Supplement: Additional file 11 — Waterfall and boxplot analysis's shows a differential EMT score for colon < lung < pancreas following normalization across all samples. [file 1755-8794-4-9-S11.PDF]

# Additional File 12

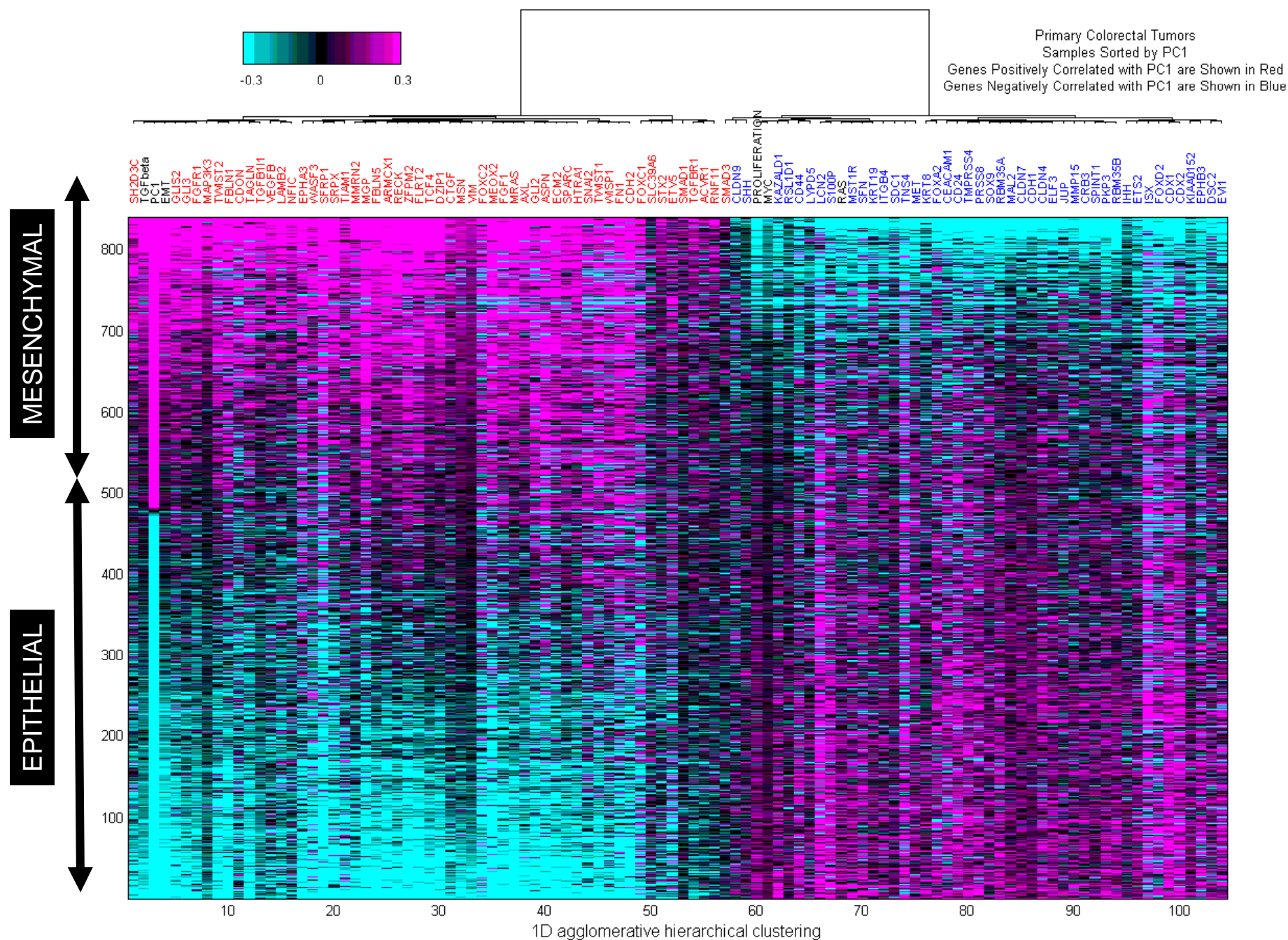

Supplement: Additional file 12 — Top 5000 most variable genes (columns) on Colon cell lines (rows) sorted by PC1. PC1 is observed to be positively correlated to EMT signature score and anti-correlated to RAS signature score. Genes are clustered using Pearson correlation distance metric and Ward linkage. Heatmap shows mean-centered probe intensities. [file 1755-8794-4-9-S12.PDF]

# Additional File 13

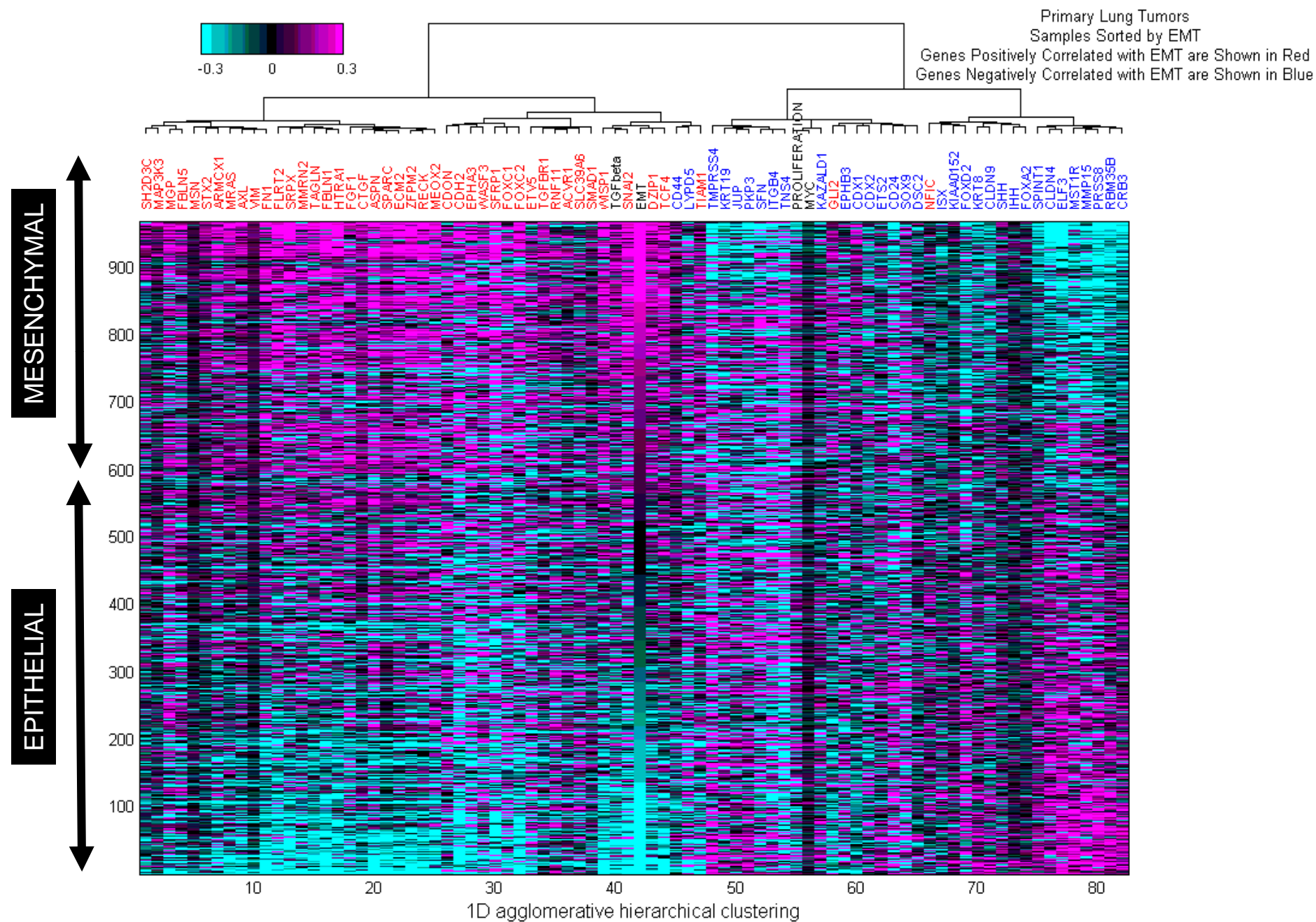

Supplement: Additional file 13 — PC1 predicts recurrence in stages 2 and 3 of colon cancer. Data is shown for MCC dataset. [file 1755-8794-4-9-S13.PDF]

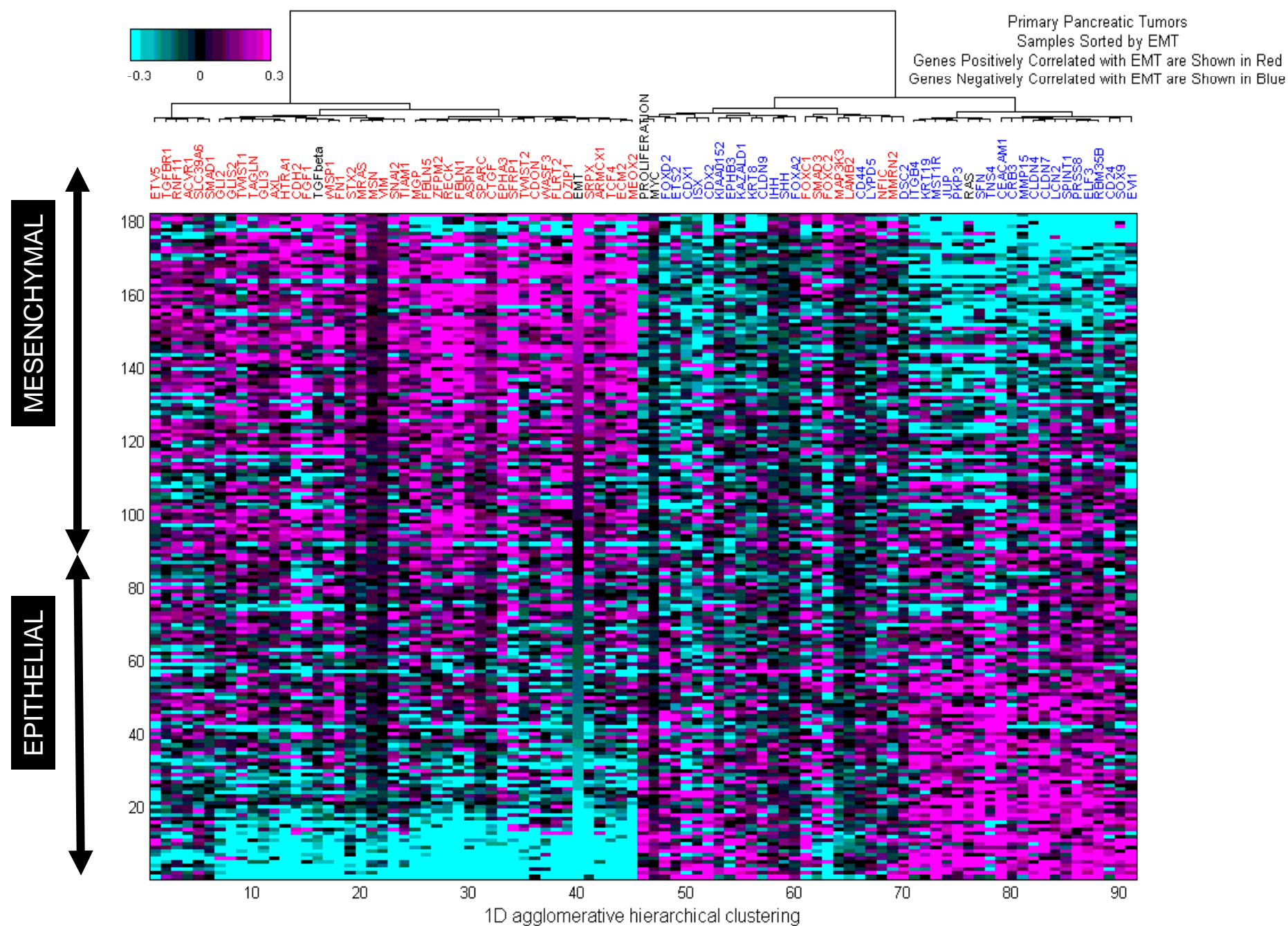

Supplement: Additional file 14 — Covariance matrices showing the relationship of PC1 to the same endpoints as shown in Figure 4ausing (a) independent colon dataset [21](b) EXPO dataset, (c) NKI dataset. [file 1755-8794-4-9-S14.PDF]

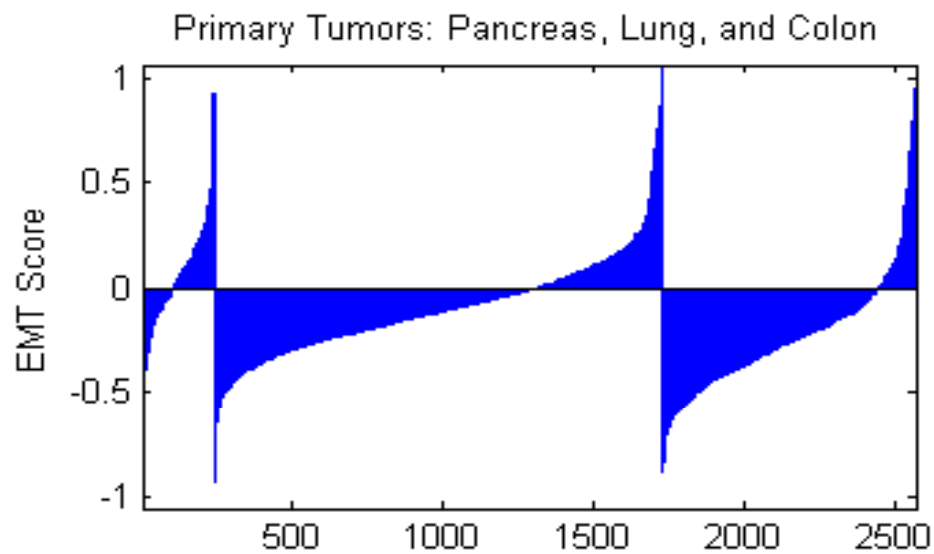

## Primary Tumors

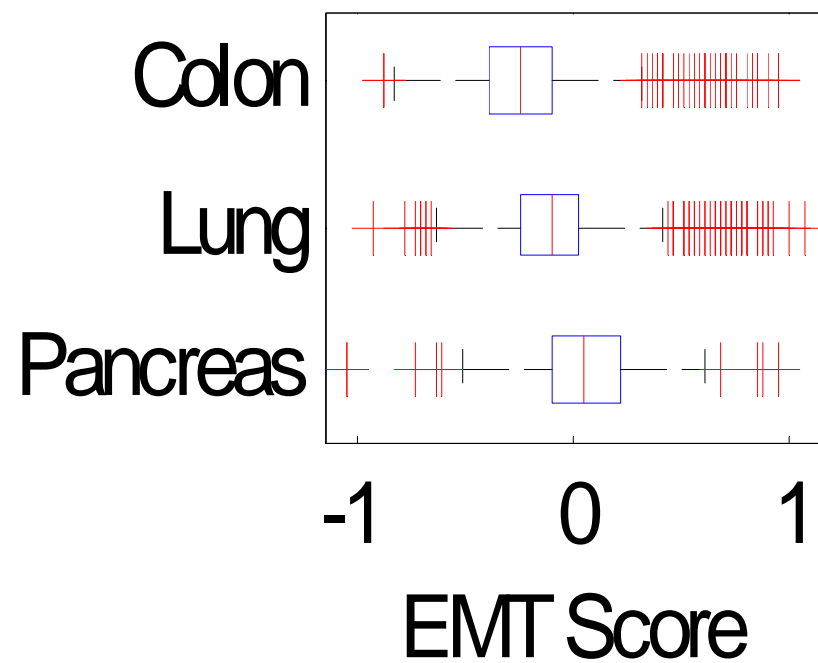

Supplement: Additional file 15 — EMT signature proposed in this paper is predictive of recurrence in stage 2 and stage 3 MCC tumors. [file 1755-8794-4-9-S15.PDF]

Additional File 16.

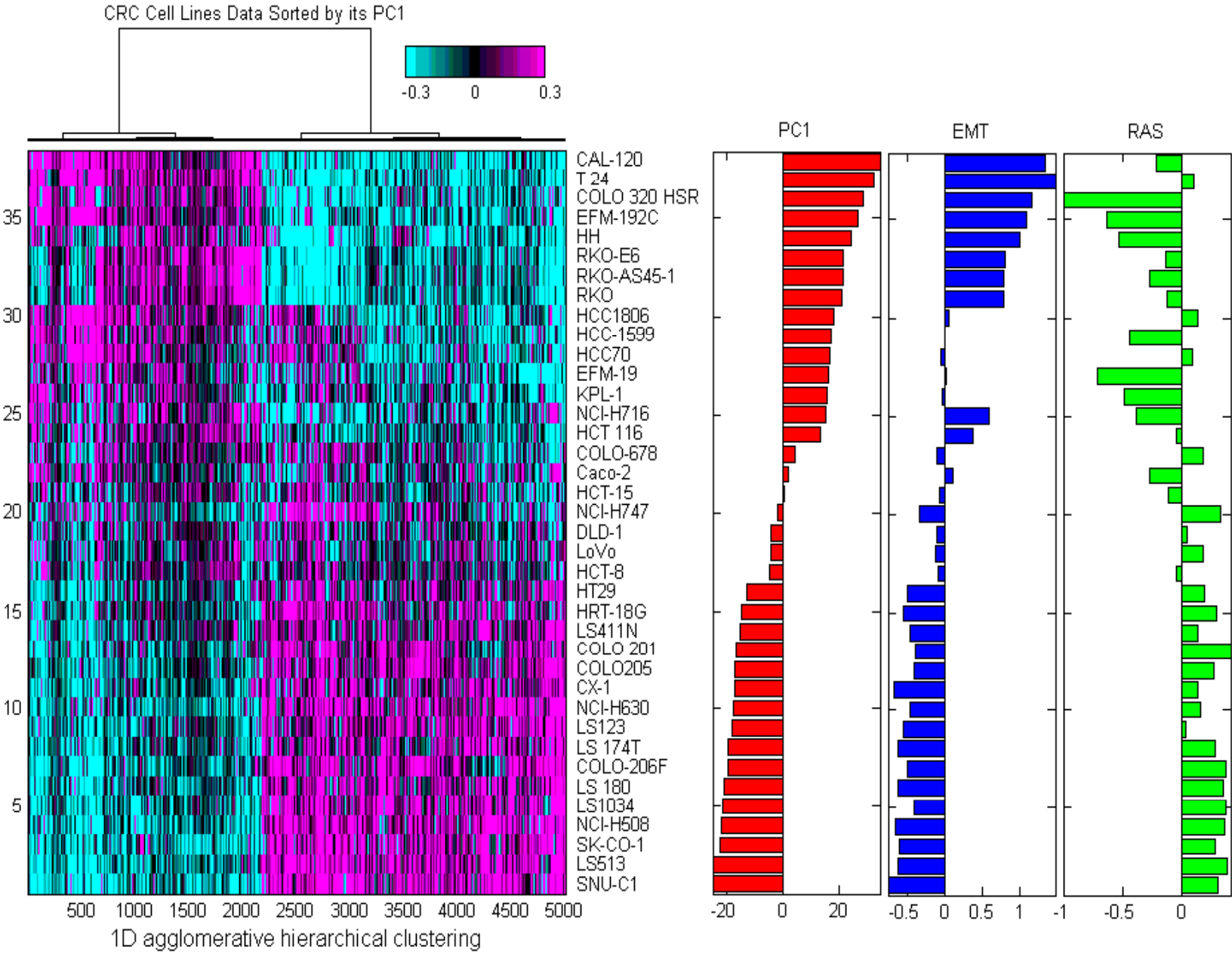

Supplement: Additional file 16 — EMT signature proposed in this paper is predictive of recurrence when applied to all tumor samples in MCC data set. [file 1755-8794-4-9-S16.PDF]
