# Supplementary material for: EMT is the dominant program in human colon cancer
Source: BMC Med Genomics. 2011 Jan 20;4:9. doi: 10.1186/1755-8794-4-9 (PMC3032646; doi:10.1186/1755-8794-4-9)
Supplement: Additional file 4 — Centered abundances for 49 tumors × 416 MiR detectors. [file 1755-8794-4-9-S4.PDF]

Additional **File 4**.

				201131_s_at	201131_s_at	212764_at	212764_at	203603_s_at	203603_s_at
				CDH1	CDH1	ZEB1	ZEB1	ZEB2	ZEB2
				Pearson	Pearson	Pearson	Pearson	Pearson	Pearson
eg.probe	eg.affy	eg_symbol	arm	corr	p-value	corr	p-value	corr	p-value
21375	201131_s_at	CDH1	EMT DOWN	100%	0.E+00	-34%	2.E-10	-25%	4.E-06
42583	224650_at	MAL2	EMT DOWN	79%	0.E+00	-36%	2.E-11	-22%	8.E-05
19647	202890_at	MAP7	EMT DOWN	76%	0.E+00	-40%	3.E-14	-39%	5.E-13
4491	218186_at	RAB25	EMT DOWN	76%	0.E+00	-44%	6.E-17	-33%	6.E-10
21135	201428_at	CLDN4	EMT DOWN	75%	0.E+00	-27%	5.E-07	-19%	4.E-04
11812	210827_s_at	ELF3	EMT DOWN	73%	0.E+00	-37%	3.E-12	-32%	4.E-09
11881	210715_s_at	SPINT2	EMT DOWN	73%	0.E+00	-34%	3.E-10	-23%	3.E-05
22890	235141_at	MARVELD2	EMT DOWN	73%	0.E+00	-47%	1.E-19	-44%	2.E-16
5807	216905_s_at	ST14	EMT DOWN	73%	0.E+00	-42%	2.E-15	-31%	7.E-09
12848	209771_x_at	CD24	EMT DOWN	72%	0.E+00	-22%	6.E-05	-25%	7.E-06
4473	218261_at	AP1M2	EMT DOWN	71%	0.E+00	-47%	1.E-19	-43%	6.E-16
12499	210058_at	MAPK13	EMT DOWN	71%	0.E+00	-34%	3.E-10	-27%	1.E-06
13905	208651_x_at	CD24	EMT DOWN	71%	0.E+00	-24%	1.E-05	-23%	2.E-05
13904	208650_s_at	CD24	EMT DOWN	71%	0.E+00	-26%	2.E-06	-23%	3.E-05
6294	216379_x_at	CD24	EMT DOWN	71%	0.E+00	-22%	6.E-05	-24%	8.E-06
21036	201510_at	ELF3	EMT DOWN	71%	0.E+00	-35%	5.E-11	-31%	6.E-09
12500	210059_s_at	MAPK13	EMT DOWN	71%	0.E+00	-36%	2.E-11	-22%	9.E-05
21916	200606_at	DSP	EMT DOWN	70%	0.E+00	-30%	5.E-08	-20%	3.E-04
50	266_s_at	CD24	EMT DOWN	70%	0.E+00	-22%	5.E-05	-24%	1.E-05
40926	226213_at	ERBB3	EMT DOWN	68%	0.E+00	-41%	2.E-14	-26%	3.E-06
20097	202454_s_at	ERBB3	EMT DOWN	68%	1.E-45	-42%	3.E-15	-23%	2.E-05
19151	203397_s_at	GALNT3	EMT DOWN	68%	3.E-45	-34%	4.E-10	-22%	5.E-05
22007	65517_at	AP1M2	EMT DOWN	68%	6.E-45	-46%	1.E-18	-45%	9.E-18
19722	202790_at	CLDN7	EMT DOWN	68%	1.E-44	-37%	3.E-12	-27%	1.E-06
12849	209772_s_at	CD24	EMT DOWN	67%	6.E-44	-28%	3.E-07	-23%	2.E-05
20535	202005_at	ST14	EMT DOWN	67%	7.E-44	-41%	1.E-14	-33%	1.E-09
20906	201650_at	KRT19	EMT DOWN	67%	2.E-43	-30%	4.E-08	-13%	2.E-02
3765	218960_at	TMPRSS4	EMT DOWN	67%	3.E-43	-41%	3.E-14	-31%	1.E-08
19677	202831_at	GPX2	EMT DOWN	66%	1.E-42	-38%	2.E-12	-27%	5.E-07
7939	214774_x_at	TOX3	EMT DOWN	66%	3.E-42	-27%	6.E-07	-18%	1.E-03
21374	201130_s_at	CDH1	EMT DOWN	66%	1.E-41	-30%	3.E-08	-26%	3.E-06
6069	216623_x_at	TOX3	EMT DOWN	66%	2.E-41	-27%	5.E-07	-18%	1.E-03
7621	215108_x_at	TOX3	EMT DOWN	65%	4.E-41	-29%	1.E-07	-19%	6.E-04
19994	202525_at	PRSS8	EMT DOWN	65%	8.E-41	-27%	1.E-06	-21%	2.E-04
28243	229842_at	ELF3	EMT DOWN	65%	1.E-40	-37%	6.E-12	-33%	9.E-10
12678	209873_s_at	PKP3	EMT DOWN	65%	1.E-40	-41%	2.E-14	-32%	2.E-09
19672	202826_at	SPINT1	EMT DOWN	65%	5.E-40	-44%	4.E-17	-36%	1.E-11
37288	239148_at	MARVELD3	EMT DOWN	65%	7.E-40	-44%	5.E-17	-39%	2.E-13
20684	201884_at	CEACAM5	EMT DOWN	64%	1.E-39	-24%	9.E-06	-13%	2.E-02
43991	223233_s_at	CGN	EMT DOWN	64%	3.E-39	-21%	1.E-04	-22%	9.E-05
2784	219946_x_at	MYH14	EMT DOWN	64%	7.E-39	-40%	1.E-13	-33%	8.E-10
20045	202489_s_at	FXYD3	EMT DOWN	64%	3.E-38	-28%	4.E-07	-20%	3.E-04
7249	215471_s_at	MAP7	EMT DOWN	64%	3.E-38	-45%	1.E-17	-37%	6.E-12
10996	211657_at	CEACAM6	EMT DOWN	63%	7.E-38	-21%	2.E-04	-6%	3.E-01

EMT sig
149 UP;
160 Down

EMT UP		EMT DOWN
ADAM23		ACPP
ADAMTS1		AGR3
AFF3		ALDH3B2
AK5		ANK3
AKAP12		ANKRD22
ALPK2		ANXA9
ANGPTL2		AP1M2
ANKRD1		AQP3
ANTXR1		ARHGAP8
ANXA6		ARHGDIB
AOX1		ATAD4
AP1S2		ATP2C2
