# Supplementary material for: EMT is the dominant program in human colon cancer
Source: BMC Med Genomics. 2011 Jan 20;4:9. doi: 10.1186/1755-8794-4-9 (PMC3032646; doi:10.1186/1755-8794-4-9)
Supplement: Additional file 5 — Out of ~300 signatures tested, EMT was the most significantly associated with PC1 in colon (P < 10-135). More importantly, the up and down arms of the EMT signature were directionally correlated with PC1 (P < 10-16, Fisher Exact Test). See Additional File 2 for list of genes. [file 1755-8794-4-9-S5.PDF]

Additional **File 5.**

49 tumors 416 miRNA, mean-centered data Pearson correlation		EMT corr	EMT p-value	CDH1 corr	CDH1 p-value	ZEB1 corr	ZEB1 p-value	ZEB2 corr	ZEB2 p-value
142	hsa-miR-212-4373087 (FAM,NFQ)	46%	8.E-04	-36%	1.E-02	33%	2.E-02	40%	4.E-03
**128**	**hsa-miR-200a-4378069 (FAM,NFQ)**	**-40%**	**4.E-03**	**10%**	**5.E-01**	**-31%**	**3.E-02**	**-36%**	**1.E-02**
45	hsa-miR-132-4373143 (FAM,NFQ)	40%	4.E-03	-32%	2.E-02	28%	5.E-02	36%	1.E-02
294	hsa-miR-517a-4395513 (FAM,NFQ)	-40%	5.E-03	-25%	9.E-02	-45%	1.E-03	-41%	3.E-03
144	hsa-miR-214-4395417 (FAM,NFQ)	39%	5.E-03	-10%	5.E-01	37%	8.E-03	23%	1.E-01
208	hsa-miR-33a*-4395247 (FAM,NFQ)	-39%	6.E-03	0%	1.E+00	-31%	3.E-02	-31%	3.E-02
331	hsa-miR-576-3p-4395462 (FAM,NFQ)	-39%	6.E-03	-19%	2.E-01	-48%	4.E-04	-38%	6.E-03
414	hsa-miR-99a-4373008 (FAM,NFQ)	38%	7.E-03	-13%	4.E-01	35%	1.E-02	24%	1.E-01
**129**	**hsa-miR-200b*-4395385 (FAM,NFQ)**	**-38%**	**8.E-03**	**3%**	**8.E-01**	**-23%**	**1.E-01**	**-29%**	**4.E-02**
20	hsa-miR-100-4373160 (FAM,NFQ)	37%	9.E-03	-10%	5.E-01	35%	1.E-02	22%	1.E-01
373	hsa-miR-671-3p-4395433 (FAM,NFQ)	37%	9.E-03	-10%	5.E-01	27%	6.E-02	11%	5.E-01
390	hsa-miR-885-5p-4395407 (FAM,NFQ)	-35%	1.E-02	-10%	5.E-01	-23%	1.E-01	-32%	3.E-02
114	hsa-miR-193b-4395478 (FAM,NFQ)	35%	2.E-02	13%	4.E-01	47%	7.E-04	28%	5.E-02
161	hsa-miR-24-4373072 (FAM,NFQ)	34%	2.E-02	-1%	9.E-01	46%	9.E-04	35%	1.E-02
34	hsa-miR-125b-1*-4395489 (FAM,NFQ	34%	2.E-02	0%	1.E+00	38%	7.E-03	31%	3.E-02
309	hsa-miR-539-4378103 (FAM,NFQ)	34%	2.E-02	2%	9.E-01	33%	2.E-02	7%	7.E-01
285	hsa-miR-506-4373231 (FAM,NFQ)	34%	2.E-02	-13%	4.E-01	28%	5.E-02	19%	2.E-01
212	hsa-miR-342-3p-4395371 (FAM,NFQ)	34%	2.E-02	-28%	5.E-02	21%	1.E-01	24%	1.E-01
127	hsa-miR-200a*-4373273 (FAM,NFQ)	-34%	2.E-02	7%	6.E-01	-20%	2.E-01	-22%	1.E-01
250	hsa-miR-429-4373203 (FAM,NFQ)	-33%	2.E-02	19%	2.E-01	-23%	1.E-01	-19%	2.E-01
408	hsa-miR-942-4395298 (FAM,NFQ)	-33%	2.E-02	-9%	5.E-01	-35%	1.E-02	-25%	8.E-02
252	hsa-miR-433-4373205 (FAM,NFQ)	33%	2.E-02	16%	3.E-01	40%	5.E-03	14%	3.E-01
267	hsa-miR-489-4395469 (FAM,NFQ)	33%	2.E-02	-19%	2.E-01	19%	2.E-01	10%	5.E-01
377	hsa-miR-744*-4395436 (FAM,NFQ)	-33%	2.E-02	-2%	9.E-01	-28%	5.E-02	-29%	4.E-02
57	hsa-miR-139-5p-4395400 (FAM,NFQ)	33%	2.E-02	17%	3.E-01	41%	3.E-03	31%	3.E-02
72	hsa-miR-147b-4395373 (FAM,NFQ)	-33%	2.E-02	14%	3.E-01	-22%	1.E-01	-17%	2.E-01
85	hsa-miR-16-1*-4395531 (FAM,NFQ)	-33%	2.E-02	2%	9.E-01	-20%	2.E-01	-11%	5.E-01
35	hsa-miR-125b-4373148 (FAM,NFQ)	32%	2.E-02	-1%	9.E-01	35%	1.E-02	18%	2.E-01
60	hsa-miR-141*-4395256 (FAM,NFQ)	-32%	2.E-02	-10%	5.E-01	-32%	2.E-02	-15%	3.E-01
