# Supplementary material for: EMT is the dominant program in human colon cancer
Source: BMC Med Genomics. 2011 Jan 20;4:9. doi: 10.1186/1755-8794-4-9 (PMC3032646; doi:10.1186/1755-8794-4-9)
Supplement: Additional file 6 — Derivation of the EMT signature used to clarify the biology characterizing PC1. The EMT signature was derived from a global gene expression analysis of 93 lung cancer cell lines first segregated by differential CDH1 and VIM expression. Right panel shows the relationship between EMT signature score and CDH1 probe intensities, the left panel shows the EMT signature score vs. VIM probe intensity. EMT signature is observed to be positively correlated to VIM and anticorrelated to CDH1. [file 1755-8794-4-9-S6.PDF]

Additional File 6

Mean-centered abundances, 49 tumors x MiR 416 detectors

order	detector / tumor	T1102A	T1213A	T1249A	T1311B	T1369A	T165A	T1700A	T1792A1	T1889A1	T1954A	T2130A	T220A	T2465G
1	RNU24-4373379 (FAM,NFQ)	-0.086	0.070	0.466	0.171	0.370	-0.259	0.069	-0.392	-0.503	-0.390	0.004	-0.448	-0.390
2	RNU43-4373375 (FAM,NFQ)	0.110	0.385	0.645	-0.222	0.219	-0.240	-0.867	-0.436	-0.966	-0.880	-0.021	-0.378	-0.509
3	RNU44-4373384 (FAM,NFQ)	-0.036	0.251	0.474	-0.032	0.156	-0.078	-0.079	-0.523	-0.590	-0.479	-0.079	-0.274	-0.367
4	RNU48-4373383 (FAM,NFQ)	-0.453	0.192	0.237	0.516	-0.181	-0.797	-0.343	-0.230	-0.394	-0.288	0.058	-0.065	0.239
5	RNU6B-4373381 (FAM,NFQ)	-0.014	0.601	1.816	0.026	0.248	-2.828	-2.955	-0.221	-3.117	-0.544	1.075	-2.936	-0.304
6	has-miR-155-4395459 (FAM,NFQ)	0.243	-0.310	0.448	0.125	-0.076	0.303	0.089	-0.133	-0.182	-0.112	-0.378	0.433	0.163
7	hsa-let-7a-4373169 (FAM,NFQ)	0.006	0.076	-0.161	0.192	0.096	-0.103	0.181	0.230	0.164	0.279	-0.030	0.227	0.115
8	hsa-let-7b-4395446 (FAM,NFQ)	0.043	-0.058	-0.064	0.302	0.057	0.090	0.063	0.319	0.125	0.161	0.059	0.238	0.082
9	hsa-let-7c-4373167 (FAM,NFQ)	-0.180	0.010	-0.191	0.479	0.197	-0.314	0.059	0.509	0.173	0.276	-0.014	0.567	0.139
10	hsa-let-7d-4395394 (FAM,NFQ)	-0.016	0.016	0.049	0.353	0.107	0.006	0.153	0.250	0.272	0.158	-0.031	0.141	0.047
11	hsa-let-7e-4395517 (FAM,NFQ)	0.146	0.220	0.126	0.285	0.035	0.077	0.310	-5.955	0.356	0.246	0.110	0.242	0.235
12	hsa-let-7f-1*-4395528 (FAM,NFQ)	-0.678	-0.948	0.406	-0.744	1.505	-0.406	-0.533	0.520	-0.695	-0.671	-0.890	-0.514	-0.733
13	hsa-let-7f-2*-4395529 (FAM,NFQ)	0.127	0.603	0.109	-0.908	-0.297	0.122	-1.478	-0.723	0.026	-0.020	0.619	-1.459	-1.678
14	hsa-let-7f-4373164 (FAM,NFQ)	0.084	0.160	0.058	0.146	0.079	-0.086	0.286	0.277	0.385	0.258	0.069	-0.068	0.107
15	hsa-let-7g*-4395229 (FAM,NFQ)	0.197	0.088	0.889	-0.292	0.180	-0.081	0.284	-0.444	-0.821	-0.183	0.082	0.097	-0.560
16	hsa-let-7g-4395393 (FAM,NFQ)	0.059	0.073	0.028	-0.026	-0.166	-0.007	0.150	0.126	0.220	0.216	-0.088	-0.116	0.066
17	hsa-let-7i*-4395283 (FAM,NFQ)	-0.009	-0.092	0.404	-0.366	-0.203	0.348	-0.467	-0.056	0.009	-0.046	0.017	-0.401	-0.547
18	hsa-miR-1-4395333 (FAM,NFQ)	0.276	0.477	0.001	-0.742	-0.148	-2.867	0.440	0.921	0.432	0.840	0.536	0.590	0.552
19	hsa-miR-100*-4395253 (FAM,NFQ)	0.274	-0.150	-0.613	-0.229	0.476	0.205	0.236	-0.236	0.354	-0.123	0.101	-0.077	0.052
20	hsa-miR-100-4373160 (FAM,NFQ)	-0.191	-0.174	-0.098	0.115	0.157	-0.153	0.024	0.305	0.038	0.104	-0.145	0.291	0.154
21	hsa-miR-101-4395364 (FAM,NFQ)	0.165	0.162	-0.028	-0.021	-0.162	0.261	0.146	0.220	0.460	0.260	-0.185	-0.226	0.031
22	hsa-miR-103-4373158 (FAM,NFQ)	-0.012	0.028	0.107	0.066	0.016	0.078	0.188	0.113	0.050	0.185	-0.126	0.110	0.271
23	hsa-miR-106a-4395280 (FAM,NFQ)	-0.074	0.150	-0.051	0.348	-0.254	0.110	0.383	-0.203	0.257	0.312	-0.064	0.005	0.108
24	hsa-miR-106b*-4395491 (FAM,NFQ)	-0.150	0.261	0.521	-0.428	0.342	0.167	-1.149	-1.271	-0.077	-1.189	0.249	-1.950	-1.551
25	hsa-miR-106b-4373155 (FAM,NFQ)	0.027	0.007	0.025	0.093	-0.116	0.202	-0.164	0.040	0.242	0.250	-0.283	-0.118	0.111
26	hsa-miR-107-4373154 (FAM,NFQ)	-0.197	0.337	-0.254	0.045	-0.679	-0.265	0.039	-0.103	0.675	0.025	0.173	0.405	0.326
27	hsa-miR-10a*-4395399 (FAM,NFQ)	0.525	0.568	0.262	0.196	0.012	0.284	0.669	0.102	-0.451	0.549	-0.048	-0.007	0.081
28	hsa-miR-10a-4373153 (FAM,NFQ)	0.007	0.283	0.084	0.023	-0.022	0.207	0.348	0.061	-0.421	0.344	-0.126	0.121	0.270
29	hsa-miR-10b*-4395426 (FAM,NFQ)	0.111	0.123	0.133	-0.218	-0.416	0.151	-0.094	0.231	-0.037	-0.239	0.091	-0.083	0.577
30	hsa-miR-10b-4395329 (FAM,NFQ)	0.028	0.040	0.078	-0.268	-0.173	0.317	-0.294	0.414	-0.366	-0.219	0.006	0.159	0.782
31	hsa-miR-124-4373295 (FAM,NFQ)	0.442	0.178	1.374	0.386	-1.843	-0.843	-2.245	0.510	-2.369	0.359	0.337	-1.762	-0.049
32	hsa-miR-125a-3p-4395310 (FAM,NFQ)	0.063	-0.428	0.048	-0.175	0.112	-0.110	-0.256	-0.058	0.143	0.074	0.086	0.438	0.150
