# Supplementary material for: EMT is the dominant program in human colon cancer
Source: BMC Med Genomics. 2011 Jan 20;4:9. doi: 10.1186/1755-8794-4-9 (PMC3032646; doi:10.1186/1755-8794-4-9)

Additional **File 7**.

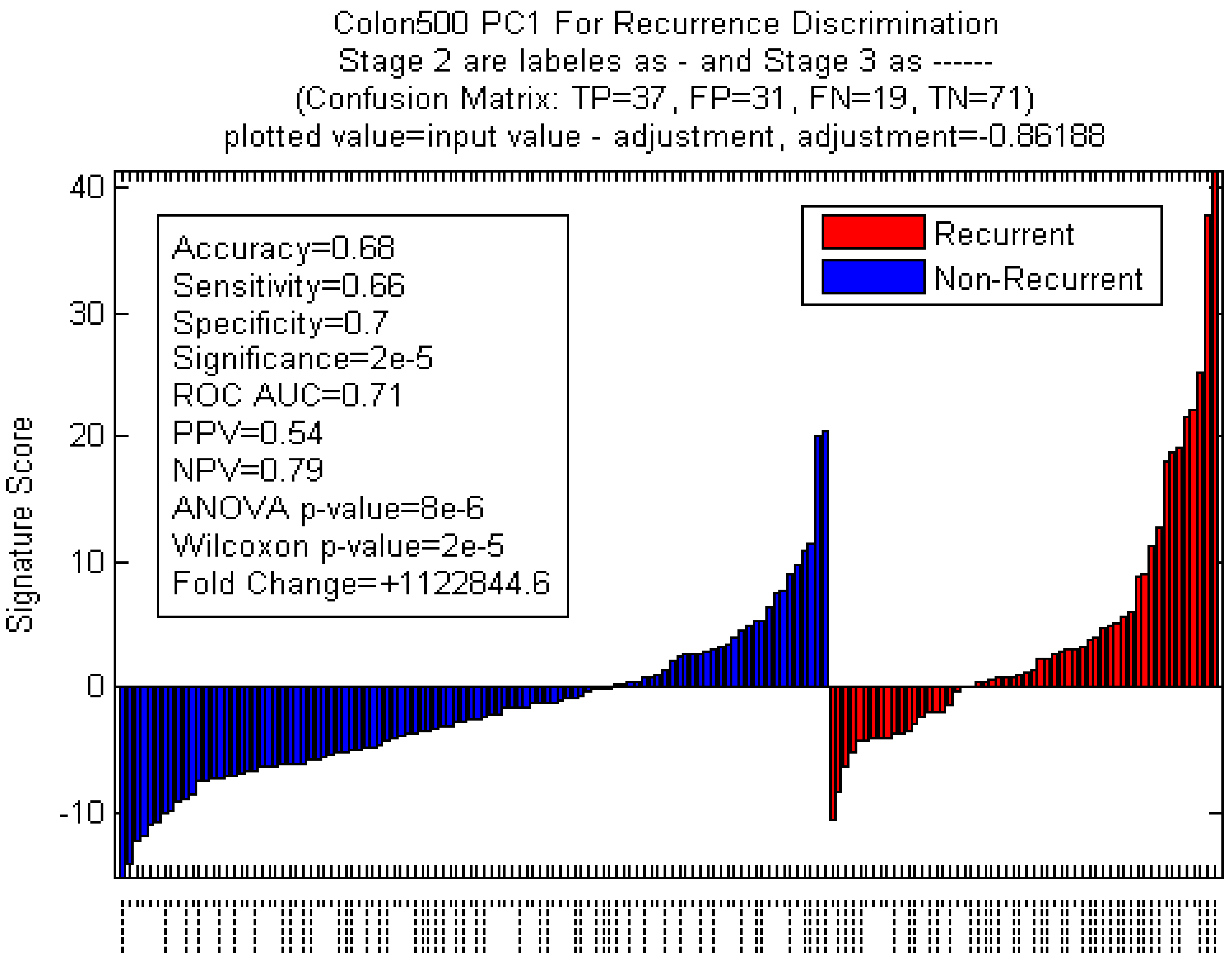

Supplement: Additional file 7 — Waterfall plot of recurrence prediction of PC1 for the MCC colon dataset shows more recurrences with high signature scores than with low signature scores; similarly there fewer recurrences with low signature scores than with high signature scores. [file 1755-8794-4-9-S7.PDF]
